# Extracellular vesicles from adipose stem cells ameliorate allergic rhinitis in mice by immunomodulatory

**DOI:** 10.3389/fimmu.2023.1302336

**Published:** 2023-12-08

**Authors:** Wenhan Yang, Zhiyu Pan, Jiacheng Zhang, Lian Wang, Ju lai, Shican Zhou, Zhili Zhang, Kai Fan, Dan Deng, Zhengliang Gao, Shaoqing Yu

**Affiliations:** ^1^Department of Otorhinolaryngology Head and Neck Surgery, Tongji Hospital, School of Medicine, Tongji University, Shanghai, China; ^2^School of Medicine, Tongji University, Shanghai, China; ^3^Department of Dermatology, Shanghai Children’s Medical Center, School of Medicine, Shanghai Jiao Tong University, Shanghai, China; ^4^Department of Dermatology, Xinhua Hospital, School of Medicine, Shanghai Jiao Tong University, Shanghai, China; ^5^Fundamental Research Center, Shanghai YangZhi Rehabilitation Hospital (Shanghai Sunshine Rehabilitation Center), School of Medicine, Tongji University, Shanghai, China; ^6^Shanghai Engineering Research Center of Organ Repair, School of Medicine, Shanghai University, Shanghai, China; ^7^Institute of Geriatrics (Shanghai University), Affiliated Nantong Hospital of Shanghai University (The Sixth People’s Hospital of Nantong), School of Medicine, Shanghai University, Nantong, China; ^8^Department of Allergy, Tongji Hospital, School of Medicine, Tongji University, Shanghai, China

**Keywords:** allergic rhinitis, extracellular vesicles, human adipose tissue-derived stem cells, anti-inflammation, Th1 and Th2 cells

## Abstract

**Background:**

Human adipose tissue-derived stem cells (hADSCs) exert potent immunosuppressive effects in the allogeneic transplantation treatment. In mouse model of allergic rhinitis (AR), ADSCs partially ameliorated AR. However, no study has evaluated the potential therapeutic effects of hADSC-derived extracellular vesicles (hADSC-EVs) on AR.

**Methods:**

Female BALB/c mice were sensitized and challenged with ovalbumin (OVA) to induce AR. One day after the last nasal drop, each group received phosphate buffered saline (PBS) or hADSC-EVs treatment. Associated symptoms and biological changes were then assessed.

**Results:**

hADSC-EV treatment significantly alleviated nasal symptoms, and reduced inflammatory infiltration. Serum levels of OVA-specific IgE, interleukin (IL)-4 and interferon (IFN)-γ were all significantly reduced. The mRNA levels of IL-4 and IFN-γ in the spleen also changed accordingly. The T helper (Th)1/Th2 cell ratio increased. The treatment efficacy index of hADSC-EV was higher than that of all human-derived MSCs in published reports on MSC treatment of AR. ADSC-EVs exhibited a greater therapeutic index in most measures when compared to our previous treatment involving ADSCs.

**Conclusion:**

These results demonstrated that hADSC-EVs could ameliorate the symptoms of AR by modulating cytokine secretion and Th1/Th2 cell balance. hADSC-EVs could potentially be a viable therapeutic strategy for AR. Further animal studies are needed to elucidate the underlying mechanisms and to optimize potential clinical protocols.

## 1 Introduction

Allergic rhinitis (AR) is a global health problem that affects over 400 million people worldwide, with a prevalence of up to 40% in some countries (1–3). AR contributes to inefficient working, sleep disturbance, and the avoidance of outdoor activities (4–6). Despite spectacular advances in the pathogenesis and treatment of AR in recent decades, the incidence of AR continues to rise, suggesting that further improvements are needed in the field (7). Specifically, there are no drugs or technologies available to completely cure AR (8). Avoidance, medications such as intranasal corticosteroids, combination therapy with antihistamines and antileukotrienes, and allergen-specific immunotherapy (AIT) are the current therapies available for AR treatment (9, 10). However, despite all these existing strategies, the disadvantages of avoiding certain activities, the lack of effect of symptomatic interventions on the immune environment to cure AR, the time-consuming nature of AIT, low compliance as well as side effects and the persistence of clinical symptoms cannot be ignored. Therefore, developing an effective cure that re-establishes the immune microenvironment quickly, eliminates symptoms meaningfully, prevents complications and minimizes side effects as much as possible is greatly needed.

AR is an upper airway disease with IgE-mediated immune responses involving sensitization to allergens mediated by antigen-presenting cells, mast cell-mediated IgE cross-linking and release of allergen molecules, inflammatory cell infiltration and nasal symptoms such as nasal itching, sneezing and rhinorrhea (2, 11). AR is typically classified as a type 2 inflammatory overt allergic disease, mainly caused by group 2 innate lymphoid cells (ILC2s), Th2 cells, dendritic cells (DCs) and epithelial cells (12). ILC2 contribute to the disease features of AR by secreting Th2-type cytokines (13). Myeloid dendritic cells (mDCs) could activate ILC2s to produce Th2 cytokines (14). Th2 cells promote IgE synthesis by secreting type 2 inflammatory cytokines such as IL-4, IL-5, IL-6, IL-9, IL-10, and IL-13. At the same time, Th1 and Th2 cells antagonize each other in AR (15). Studies have found that the balance of Th1/Th2 cells plays a crucial role in the regulation of AR immune microenvironment (2). With the deepening of AR research, more and more T cell subsets have been found to be implicated in in AR pathogenesis, such as Th9, Th17, Th22 and regulatory T (Treg) cells (16, 17). The balance of Th17 and Treg cells is also strongly associated with the development of AR (18–20).

EVs are particles with lipid bilayer structure released from various cells and play an important role in the transmission of information between nearby or distant cells (21–23). MSCs have shown exciting results in AR treatment by regulating the immune microenvironment and balancing Th1/Th2 cells through a mechanism involving EVs as well as intercellular contact (24). MSCs-EVs modulate DC function to suppress Th2 responses *in vitro* (25, 26). MSC-EVs have a number of advantages over MSCs, such as a higher level of security (27, 28). MSC-EVs are characterized by the absence of MHC class II and costimulatory molecules, which effectively avoids strong allogenic immune responses (29). MSC-EVs express several adhesion molecules, such as CD29, CD44 and CD73, which enable their homing to the inflamed tissues (30). MSC-EV prevented stem cell-induced ectopic tumour formation and immune rejection (28). MSC-EVs could be preserved by using lyophilization, which makes it easier to store and transport than MSC (31). These characteristics indicate that MSC-EVs may be a better candidate for AR treatment. However, no *in vivo* studies have yet evaluated the potential therapeutic effects of hADSC-EVs on AR (32, 33). We were the first to use untreated ADSC-EVs for the treatment of allergic rhinitis in mice. In this study, we aimed to investigate whether hADSC-EVs are an effective cell-free therapy in an ovalbumin-induced murine AR model.

## 2 Materials and methods

### 2.1 Cell culture

hADSCs were a kind gift from YH Shi (Tongji University, China) (34). hADSCs were cultured in DMEM/F12 medium (Gibco, New Mexico, USA) with 10% foetal bovine serum (FBS) (Gibco; New Mexico, USA), 100 units/mL penicillin−streptomycin (Gibco, New Mexico, USA) and 40 ug/mL Basic fibroblast growth factor (Proteintech, Wuhan, CHINA). FBS was centrifuged at 130000 × g for 18 hours at 4°C prior to use (35, 36). The cells were cultured in a 5% CO_2_ humidified atmosphere at 37°C, and the medium was changed every 3 days. After attaining 80% confluency, hADSCs were detached from the 10 cm dish using 0.05% trypsin-EDTA (Gibco, New Mexico, USA) and replated.

#### 2.1.1 hADSC characterization

Identification and characterization of hADSCs were performed with fluorescence activated cell sorting (FACS) analysis, and adipogenic differentiation and osteogenic differentiation assays were performed as previously reported (34, 37). Fourth-passage hADSCs were tested by flow cytometry analysis. Briefly, hADSCs obtained after 4 passages were incubated with fluorescence-conjugated antibodies (APC-CD73, FITC-CD90, PE-CD34, PE-CD11b, PE-CD19, PE-CD45, PE-HLA-DR) and analysed using a flow cytometer (CytoFLEX LX, BECKMAN COULTER Life Science, USA).

For adipogenic and osteogenic differentiation, ADSCs were cultured with adipogenic and osteogenic differentiation induction medium for 21 days. The differentiated cells were fixed with 4% paraformaldehyde. The adipogenic hADSCs were stained with Oil Red O (Shenggong Biotechnology (Shanghai) Co., Ltd, Shanghai, China), and the osteogenic hADSCs were stained with Alizarin Red S (Shenggong Biotechnology (Shanghai) Co., Ltd, Shanghai, China). Images were observed by a Nikon ECLIPSE Ti microscope (Nikon, Tokyo, Japan).

### 2.2 Collection and characterization of EVs

#### 2.2.1 EVs extraction and characterization

The culture medium of hADSCs between passages 4 and 6 was used to collect hADSC-EVs. hADSC-conditioned medium was collected from approximately 80% confluent hADSCs. The medium was centrifuged at 300 × g for 15 min and 4000 × g for 15 min at 4°C to further remove cells and dead cells. Then, the samples were centrifuged at 10000 × g for 30 min at 4°C to remove cellular debris by a High Speed Bench-top Centrifuge (Neofuge160R, Shanghai Lishen Scientific Instrument Co., Ltd, China), and the supernatants were ultracentrifuged at 130000 × g for 90 min at 4°C using an Optima XPN-100 (BECKMAN COULTER Life Science, USA). The precipitate was collected and suspended in PBS for immediate use.

#### 2.2.2 Transmission electron microscopy (TEM) imaging

EVs were then characterized by transmission electron microscopy as follows: hADSC-EV samples were dropped onto a copper net and incubated at room temperature for 10 min, and the excess liquid was absorbed by cleaning the absorbent paper with sterile distilled water. Ten microlitres of 2% uranyl dioxyl acetate was dropped onto the copper net for negative staining for 1 min. The suspended sample was absorbed by filter paper and dried for 2 min under an incandescent lamp. The copper mesh was observed under a transmission electron microscope (HITACHI, Tokyo, Japan) and imaged at 80 kV.

#### 2.2.3 Nanoparticle tracking analysis (NTA)

The hADSC-EV solution was diluted in PBS to a concentration range of 1 × 10^7^ - 1 × 10^9^ particles/mL. The concentration of EVs and their size and mass were measured by a particle matrix (Matric, Mel Busch, Germany) under 405 nm emission light.

#### 2.2.4 Western blot

The protein concentration of hADSC-EVs was quantified using the Pierce™ BCA protein assay (Thermo Fisher Scientific, Massachusetts, USA). Western blotting was performed as described previously (38, 39). The hADSC-EVs were analysed by Western blotting using the following primary antibodies: CD63 (1:250) (Santa Cruz Biotechnology, California, USA), HSP70 (1:1000), CD81 (1:1000), and calnexin (1:1000) (Abcam, Cambridge, UK). The figures were obtained using an imaging analysis system (GE Health Care Life Science, Uppsala, Sweden).

### 2.3 Animal preparation

Female BALB/c mice (Shanghai Jiesijie Laboratory Animal Co., Ltd., Shanghai, China) were used and bred in a specific pathogen-free (SPF) animal facility. All animal care and experimental procedures were performed in accordance with the guidelines of the National Institutes of Health guidelines and approved by the Institutional Animal Research Ethics Committee of Tongji University.

### 2.4 OVA-induced AR and therapeutic protocols

Allergic rhinitis mice were sensitized using OVA (Sigma−Aldrich, St. Louis, USA) and aluminium hydroxide (Alum) (Pierce Chemical Co. Rockford, USA). Briefly, mice were sensitized using a previously described protocol by intraperitoneal injection (IP) of 200 μL OVA solution (200 μg OVA emulsified in 20 mg of alum in a total volume of 200 μL) (40, 41). The OVA solution was used to sensitize the mice 3 times on day 1 and day 15. 10 μL OVA solution (containing 400 μg OVA emulsified with a total volume of 10 μL) was administered bilaterally via intranasal route. The OVA solution was used to challenge the mice 7 times on days 22 to 28.

**Figure 1:**
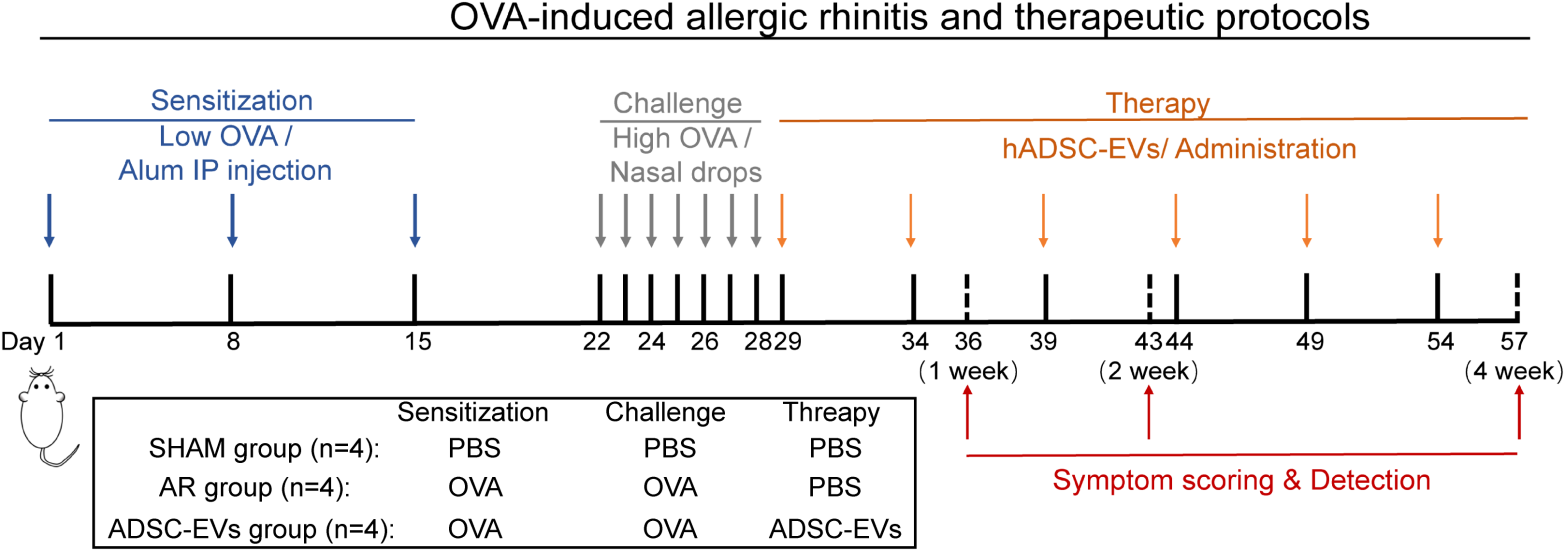
The animal experimental protocol. Mice were sensitized on days 1, 8, and 15 by an intraperitoneal injection of OVA with aluminium hydroxide. From days 22 to 28, the mice were challenged with OVA intranasal instillation using a micropipette. ADSC-EVs were injected via the tail vein on days 29, 34, 39, 44, 49, and 54, and three groups of mice were euthanized on days 36, 43, and 57.

Animals in the sham group received saline instead of OVA during the sensitization and challenge steps. Twenty female BALB/c mice were randomly divided into three groups. They were as follows: sham group (n = 4), AR group (n = 4), and hADSC-EV treatment group (n = 12). Twelve female BALB/c mice treated with hADSC-EVs were randomly divided into three groups (n = 4): 1-week treatment group, 2-week treatment group, and 4-week treatment group. One day after the last challenge, the hADSC-EV treatment groups were injected with hADSC-EVs via the tail vein. hADSC-EVs were administered every five days during treatment. In the treatment group, the hADSC-EV dose was administered through the tail vein as follows: 0.5 × 10^6^ cell secretions, 200 μL (**Figure 1**).

1-week treatment group: hADSC-EVs were administered 2 times on days 29 and 34.
2-week treatment group: hADSC-EVs were administered 3 times on days 29, 34, and 39.
4-week treatment group: hADSC-EVs were administered 6 times on days 29, 34, 39, 44, 49, 54, and 57.

Mice in the 1-week group, 2-week group, and 4-week group were euthanized on days 36, 43, and 57, respectively.

### 2.5 Characterization of nasal allergic symptoms

Sneezing and nasal rubbing were characterized. In detail, the characterization method was as follows: On the day of the last challenge, the sum of nasal scratching and sneezing events of each mouse was recorded within 15 min. After the above treatments, the sham group was stimulated with PBS, and the model group and treatment groups were stimulated with OVA before euthanasia. The sum of nasal scratching and sneezing events was then observed and recorded within 15 min after stimulation. The frequency of sneezing and nasal rubbing was recorded blindly by two observers.

### 2.6 Histologic analysis and immunohistochemistry

After the mice were euthanatized by cervical dislocation, the nasal mucosa was collected and washed with PBS, fixed with 4% paraformaldehyde and embedded in paraffin wax. The paraffin blocks of nasal mucosa were sectioned serially and stained with an haematoxylin and eosin (H&E) and periodic acid-Schiff (PAS) Staining Kit (Wuhan Xavier Biotechnology Co., Wuhan, China). Finally, images were collected and analysed on a digital section scanner (Pannoramic 250FLASH, 3DHISTECH, Hungary).

### 2.7 Measurement of serum OVA-specific IgE, OVA-specific IgG and cytokines

After symptom scoring, blood samples of mice were immediately collected via eyeball, clotted at room temperature for 20 min and centrifuged at 2000 × g for 20 min to extract serum. The levels of OVA-specific IgE, OVA-specific IgG, IL-4, IFN-γ and TGF-β1 were measured by an enzyme-linked immunosorbent assay (ELISA) kit (AMEKO, Shanghai, China) in accordance with the manufacturer’s instructions.

### 2.8 Quantitative real-time polymerase chain reaction (qRT−PCR) analysis

Fresh spleens of mice were separated under aseptic conditions after euthanasia. Total RNA was extracted from the spleen using the TRIzol reagent kit (Invitrogen, California, USA). Equivalent amounts of RNA were reverse transcribed using the one-step 1st Strand cDNA Synthesis SuperMix (Novoprotein, Suzhou, CHINA). The mRNA expression analysis was performed using the QuantStudio 5 Real Time PCR System (Thermo, Massachusetts, USA). The corresponding primers are shown in following.

β-actin: Froward primer 5’- GGCTGTATTCCCCTCCATCG -3’ and Reverse primer 5’- CCAGTTGGTAACAATGCCATGT -3’.
IL-4: Froward primer 5’- GGTCTCAACCCCCAGCTAGT -3’ and Reverse primer 5’- GCCGATGATCTCTCTCAAGTGAT -3’.
IFN-γ: Froward primer 5’- ATGAACGCTACACACTGCATC -3’ and Reverse primer 5’- CCATCCTTTTGCCAGTTCCTC -3’.
IL-17: Froward primer 5’- TCTCTGATGCTGTGTTGCTGCT -3’ and Reverse primer 5’- CGTGGAACGGTTGAGGTAGT -3’.

The average transcript levels of genes were normalized to β-actin levels. The relative expression levels of target genes were calculated by the 2^-ΔΔCT^ method.

### 2.9 Flow cytometry analysis

Mouse spleen lymphocytes were isolated with mouse lymphocyte isolation solution (Dakewe Biotech Co., Ltd., Shenzhen, China) and stimulated with Cell Activation Cocktail (Biolegend, California, USA). Then, lymphocytes were blocked with anti-mouse CD16/32 and stained with FITC anti-mouse CD4 antibodies, and membrane rupture was performed with buffer. Finally, APC-IL-4, and Brilliant Violet 421-IFN-γ were added for intracellular staining. Data were acquired using a Cytoflex LX flow cytometer (Beckman Coulter, Brea, USA) and analysed using FlowJo software (Becton, Dickinson and Company, Lake Franklin, USA).

### 2.10 Treatment effectiveness index analysis

The databases PubMed, Web of Science and Google Scholar were searched from their creation to June 2023 included. Eight groups of term were searched, including extracellular vesicle & nasal (132 publications), Exosomes & rhinitis (33 publications), extracellular vesicles & rhinitis (42 publications), exosomes & rhinosinusitis (25 publications), extracellular vesicles & rhinosinusitis (27 publications) Adipose stem cell derived vesicles (534 publications), Adipose stem cell derived vesicles & rhinitis (0 publication) Adipose stem cell derived exosomes & rhinitis (0 publication).

Articles on the treatment of allergic rhinitis mouse models with mesenchymal stem cells from different sources were screened and the original data were retrieved from the original text or the data were obtained after processing the articles using ImageJ software (National Institutes of Health, Maryland, USA). Data from the articles that met the requirements were extracted and analysed using the Treatment Effectiveness Index (TEI).

### 2.11 Statistical analysis

Results were shown as the mean ± standard deviation (SD). Multiple groups were compared with analysis of variance (ANOVA) or Student’s t-test using SPSS software 26.0 (SPSS Inc., Chicago, USA). Results with a p-value of< 0.05 were considered significant. Statistical results were performed by using GraphPad Prism version 8 (GraphPad Software, San Diego, USA).

## 3 Results

### 3.1 Collection and characterization of EVs from hADSCs

**Figure 2:**
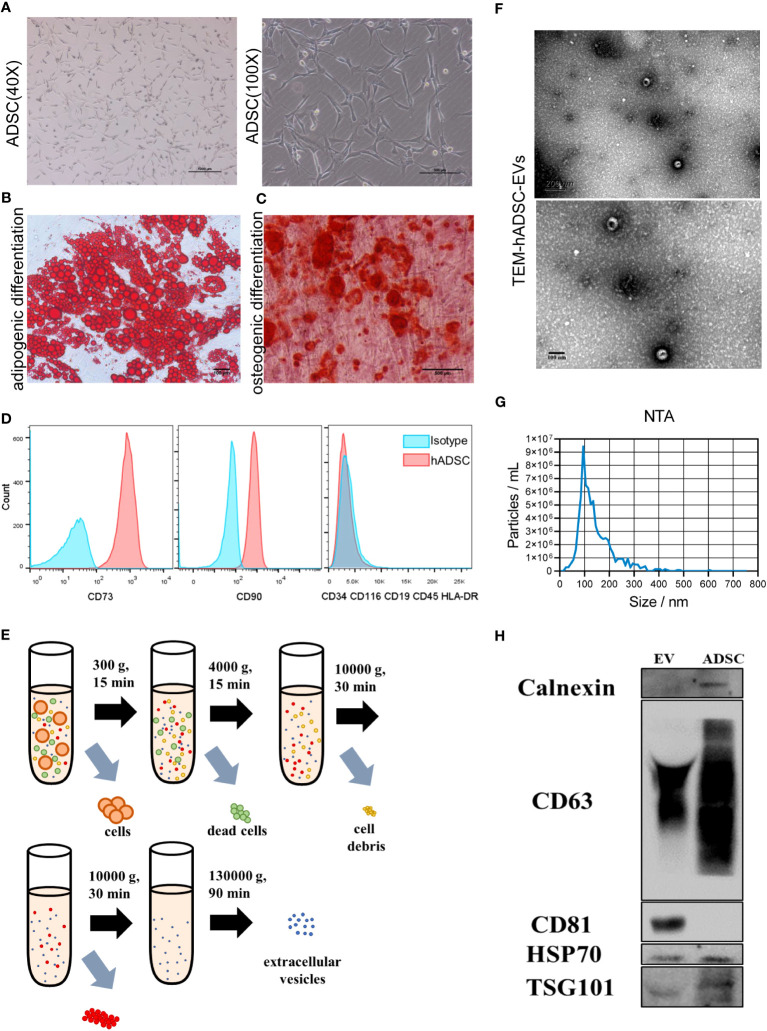
Characterization of hADSCs and hADSC-EVs. **(A)** Phase contrast image of hASDSCs in the P4 generation. **(B, C)** Representative images of adipogenic hASDSCs by oil red O staining and osteogenic cells by alizarin red staining. **(D)** Flow cytometry analysis of surface antigen expression on cultured hADSCs. **(E)** Schematic process of isolation and purification of extracellular vesicles. **(F)** Representative image of EVs by transmission electron microscopy (TEM). **(G)** Particle size distribution of EVs measured by nanoparticle tracking analysis (NTA). **(H)** Results of Western blot analysis of specific surface markers.

hADSCs were observed to adhere to cell culture plates (**Figure 2A**) and exhibited multilineage differentiation processes, including adipogenesis (**Figure 2B**) and osteogenesis (**Figure 2C**). hADSCs were positive for CD73 and CD90 and did not express negative markers CD34, CD116, CD9, CD45 and HLA-DR (**Figure 2D**). Culture medium from passage 4-6 hADSCs was harvested and subjected to ultracentrifugation to collect EVs (**Figure 2E**), which showed the typical bowl shape of EVs by TEM (**Figure 2F**). NTA demonstrated that the extracellular vesicles had a diameter of approximately 100 nm and a particle concentration of 8.7 × 10^7^ particles/mL at a dilution factor of 4000 (**Figures 2G**, **S1A, B**). In addition, hADSC-EVs were positive for CD81, TSG101, and CD63 and negative for calnexin, as detected by Western blot analysis (**Figure 2H**). Taken together, these results confirmed the successful collection of hADSC-EVs.

### 3.2 hADSC-EVs inhibit symptoms and inflammation

**Figure 3:**
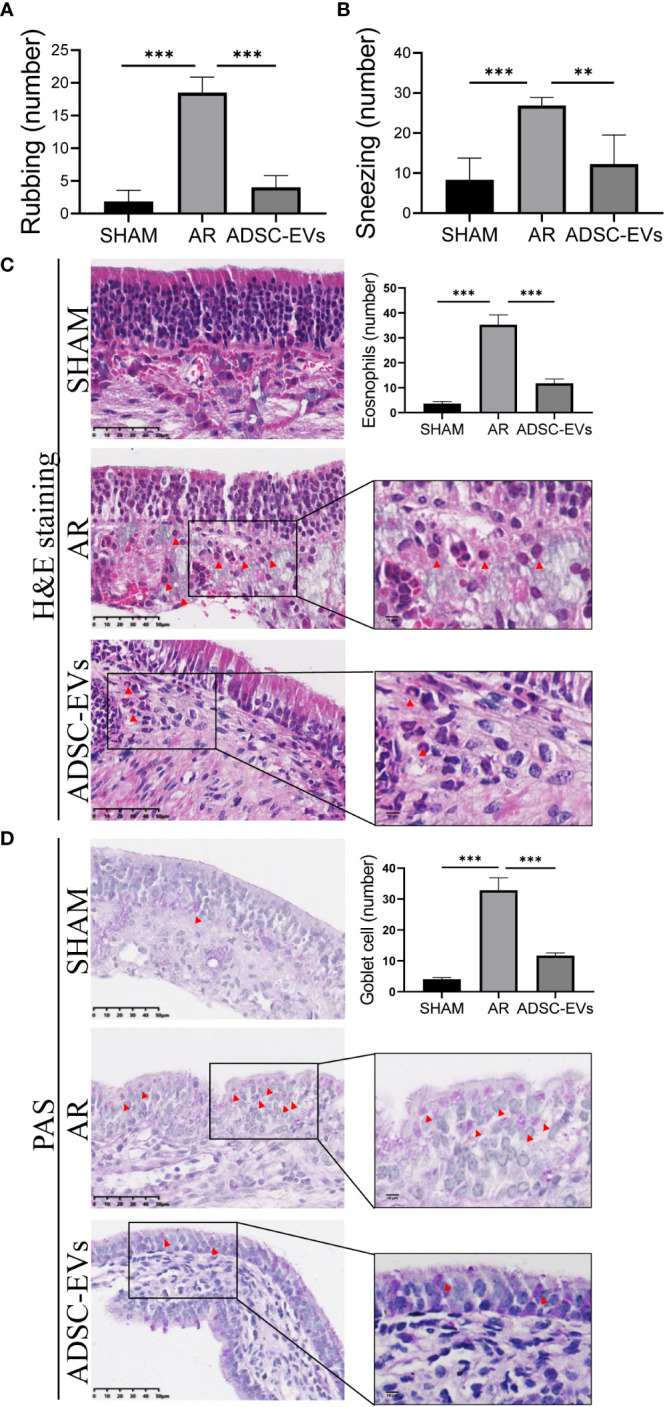
hADSC-EVs alleviated inflammation in the nasal mucosa of AR mice. **(A, B)** Quantification of sneezing and rubbing events 15 min after the final ADSC-EV administration. **(C)** H&E staining and quantification of eosinophils in the nasal mucosa. **(D)** PAS staining and quantification of goblet cells in the nasal mucosa. Data are expressed as mean ± SD, n=8 or 24, **P< 0.01, ***P< 0.001.

Nasal symptoms commonly associated with AR, such as sneezing and nasal rubbing, were analysed in different groups. The number of sneezing and nasal rubbing events in the hADSC-EV group was significantly lower than that in the AR group (**Figures 3A, B**), suggesting that this treatment has an exciting therapeutic effect on symptoms associated with AR.

Patients with AR tend to have a higher absolute eosinophil counts, and eosinophil accumulation and inflammatory cell infiltration at sites of inflammation are positively correlated with allergic diseases (42–45). The inflammatory microenvironment of the nasal mucosa was examined by histological H&E staining and PAS staining to assess tissue eosinophil infiltration, goblet cell hyperplasia and blood vessel proliferation. The study revealed that the sham group displayed a negligible number of inflammatory cells in H&E staining. On the contrary, a significant number of eosinophils infiltrated the tissue in the AR group, as illustrated by the red arrow (**Figure S2A**). Additionally, the cilia detached, while the small blood vessels proliferated and expanded (**Figure S2A**). Remarkably, the AR group showed obvious goblet cell hyperplasia, as pointed out by the black arrow (**Figure S2B**).

Based on the statistical analysis, the hADSC-EV groups exhibited significantly lower eosinophil count when compared to the AR group (**Figure 3C**). Moreover, PAS staining demonstrated a significant improvement in goblet cell enlargement and proliferation after hADSC-EV treatment (**Figure 3D**). These findings indicate that hADSC-EVs can effectively inhibit the development of allergic inflammation with regards to nasal symptoms.

### 3.3 hADSC-EVs inhibit the secretion of immunoglobulin and cytokines

**Figure 4:**
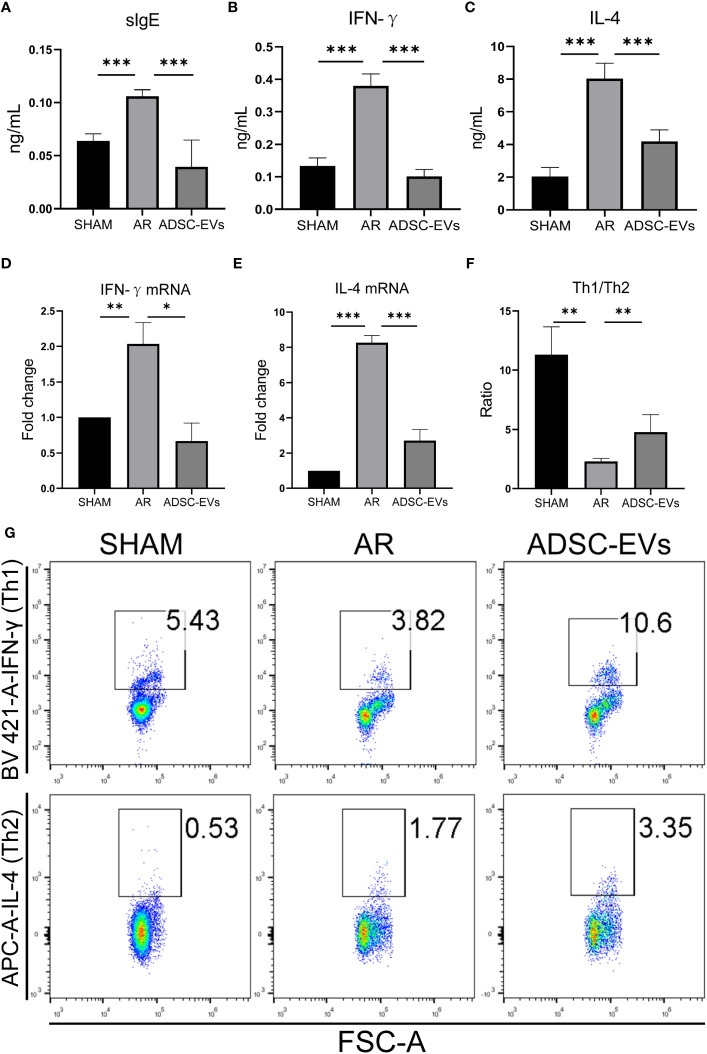
hADSC-EVs alleviated the Th1/Th2 imbalance and the secretion of antigen-specific antibodies and cytokines in AR animal models. **(A–C)** Concentration of sIgE, IFN-γ and IL-4 in the serum measured by ELISA. **(D, E)** Relative expression of IFN-γ and IL-4 by qRT−PCR. **(F)** The ratio of Th1/Th2 cells calculated according to the results from **(G)**. **(G)** Flow cytometry analysis of Th1 cells and Th2 cells in splenic lymphocytes. Data are expressed as mean ± SD, n=8, *P< 0.05, **P< 0.01, ***P< 0.001.

OVA-sIgE was found to be closely associated with symptoms such as sneezing and nasal itching in AR (2), the level of serum secreted sIgE in the AR group was higher than that in the sham group, and the level in the hADSC-EV group was obviously lower than in the AR group (**Figure 4A**). In addition, the level of IgG secreted in serum was lower in the ADSC-EV group than in the AR group (**Figure S2C**). Similar results were observed in the levels of IFN-γ and IL-4 in the serum. The highest levels were found in the AR group (**Figures 4B, C**), but they significantly decreased after hADSC-EV administration.

The relative mRNA expression of IFN-γ and IL-4 was significantly higher in the AR group when compared to the sham group. After hADSC-EV treatment, however, these mRNA expression levels substantially declined (as indicated in **Figures 4D, E**). Consequently, this finding suggests that hADSC-EV treatment could be efficacious in suppressing cytokine production in AR.

### 3.4 hADSC-EVs increase the Th1/Th2 ratio in the spleen

The role of cytokines in AR has been extensively researched. When exposed to an allergen, naive helper T cells (Th0) are activated and differentiate into Th2 cells, causing an imbalance of Th1 and Th2 (6). We used flow cytometry to investigate the Th1/Th2 cell ratio. As shown in **Figures 4F, G**, the Th1/Th2 ratio was significantly lower in the sham group than in the AR group. Interestingly, the Th1/Th2 cell ratio apparently increased after administering hADSC-EVs. Additionally, the CD4+ T cell ratio was lower in the ADSC-EV group than in the AR group, although there was no significant difference (**Figure S2D**). The Th1 cell ratio in both the hADSC-EV and sham groups was higher than that in the AR group (**Figure S2E**), while the Th2 cell ratio was the opposite (**Figure S2F**). The results indicate that hADSC-EV therapy led to an increase in the number of Th1 cells and a decrease in the number of Th2 cells to some extent. Additionally, the therapy improved the imbalance between Th1 and Th2 cells in the spleen.

### 3.5 One week of treatment can effectively relieve the allergic symptoms of allergic rhinitis mice

**Figure 5:**
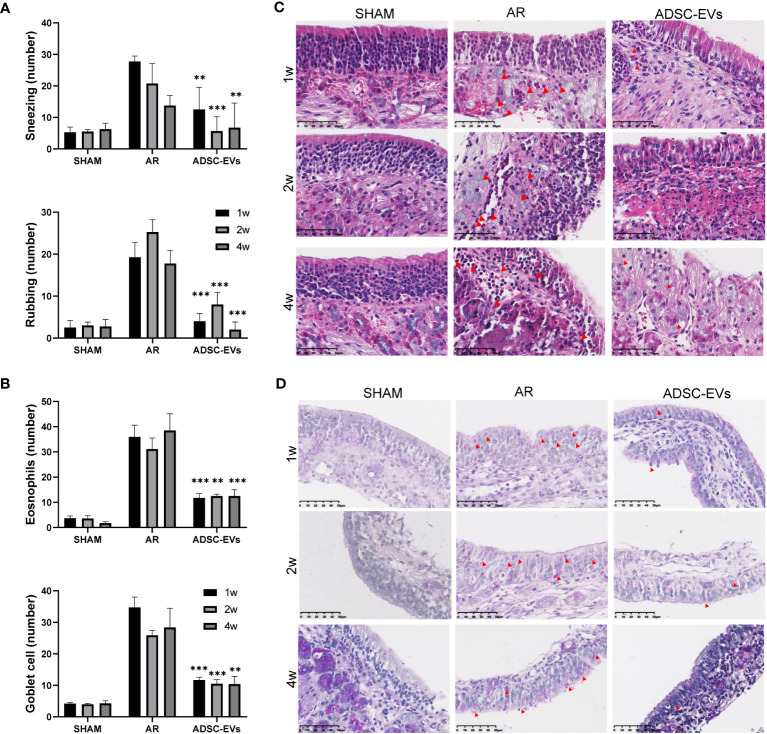
Multiple dosing durations of hADSC-EVs alleviated inflammation in the nasal mucosa of AR mice. **(A)** The number of sneezing and rubbing events 15 min after the final administration. **(B)** The count of eosinophils and goblet cells. **(C)** H&E staining of the nasal mucosa. **(D)** PAS staining of the nasal mucosa. Data are expressed as mean ± SD, n=8 or 24, **P< 0.01, ***P< 0.001.

To investigate the effect of treatment durations on outcome, three treatment lengths were designed. Analysis of the behavioural results showed that after 1, 2 and 4 weeks of treatment (**Figure 5A**), the number of sneezes and rubs were significantly lower in the treated group than in the AR group. There was no apparent difference between the three treatment periods.

According to the H&E and PAS staining results (**Figures 5B–D**), small blood vessel proliferation, eosinophil infiltration and goblet cell proliferation were alleviated in all three treatment groups, unlike in the AR group. The duration of administration did not have a significant impact on the alleviating effect.

**Figure 6:**
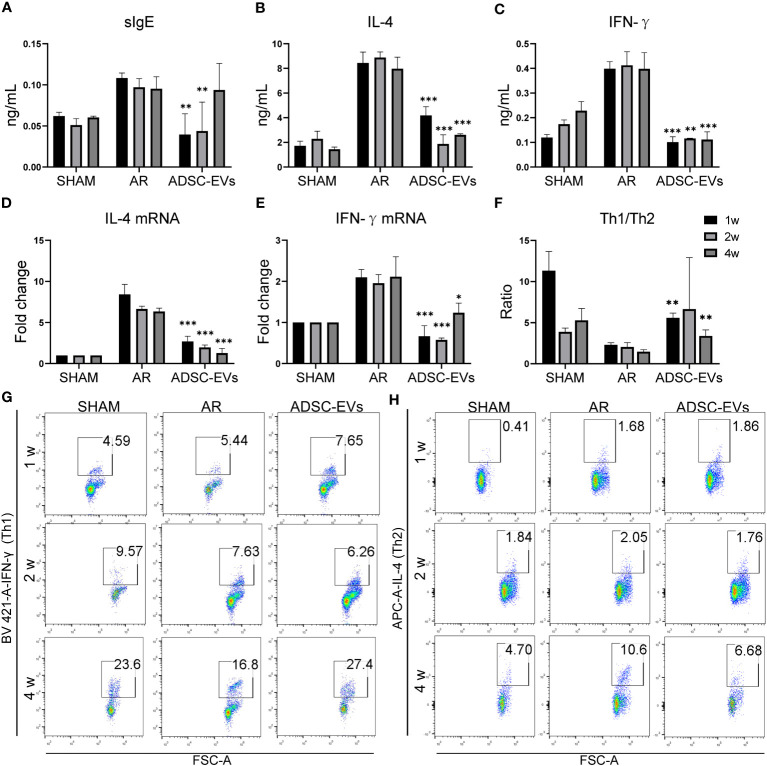
Multiple dosing durations of hADSC-EVs alleviated the Th1/Th2 imbalance and the secretion of antigen-specific antibodies and cytokines in AR animal models. **(A–C)** Serum sIgE, IL-4 and IFN-γ concentrations were measured by ELISA kits. **(D, E)** The relative mRNA expression levels of IL-4 and IFN-γ were measured by qRT−PCR. **(F)** The Th1/Th2 ratio. **(G, H)** Flow cytometry analysis of Th1 cells and Th2 cells and the Th1/Th2 cell ratio in the splenic lymphocytes of mice. Data are expressed as mean ± SD, n=8, *P< 0.05, **P< 0.01, ***P< 0.001.

The ELISA results showed that the levels of OVA-specific immunoglobulin antibodies were variable (**Figure 6A**). As administration increased, the evident decrease in sIgE secretion in the ADSC-EV group dissipated (p< 0.01, p< 0.01 and ns, respectively). The decrease in both IL-4 and IFN-γ secretion remained constant throughout the duration of administration in the ADSC-EVs group as shown in **Figure 6B**, C (IL-4: p< 0.001, p< 0.001 and p< 0.001; IFN-γ: p< 0.001, p< 0.01 and p< 0.001, respectively).

Furthermore, the decrease in IL-4 mRNA expression in the ADSC-EV group remained consistent regardless of the duration of drug administration. The expression of IFN-γ in the group that underwent 2-week treatment was lower compared to that in the 1-week and 4-week treatment groups. Moreover, it was significantly lower than the expression in the AR group (p< 0.001, p< 0.001 and p< 0.05, respectively) (**Figures 6D, E**).

Spleen lymphocytes were sorted and analyzed using flow cytometry (**Figures 6F–H**). The analysis revealed a considerable enhancement in the Th1/Th2 ratio at 1 and 4 weeks of hADSC-EV administration (p< 0.01 and p< 0.01, respectively), along with a tendency towards an increase at 2 weeks. The results clearly indicate that a one-week treatment can produce substantial therapeutic outcomes in AR mice.

### 3.6 Similar therapeutic effects were achieved using a lower ADSC-EV treatment frequency

To evaluate whether the therapeutic efficacy of ADSC-EVs administration surpasses ADSC administration in AR mouse model treatment, we conducted a comprehensive search on PubMed and Web of Science. Our analysis included 17 articles that utilized the same administration method as our study (8, 45–60). In these 17 studies, various types of MSCs such as ADSCs, umbilical cord MSCs (UC-MSCs), BMSCs, tonsil MSCs (T-MSCs), human deciduous tooth MSCs (DT-MSCs), and nasal mucosa MSCs (NM-MSCs) were administered via different dosages and administration methods (**Supporting information: Table 1**).

Due to variations in the protocols employed to establish the AR model and in the statistical methods used to analyze treatment indicators, we have developed a new parameter, namely the Treatment Effectiveness Index (TEI), based on a data -standardized approach (maximum-min Normalization) to more effectively compare differences in treatment outcomes (61). The TEI is calculated by dividing the difference between the treatment group and the AR model group by the difference between the sham group and the AR model group. The greater the TEI, the more effective the treatment.


$$\text{TEI}=\left|T_{a}-T_{t}\right|/\left|T_{a}-T_{c}\right|$$

$$T_{t}:\;the\;value\;in\;the\;treatment\;group.$$

$$T_{a}:\;the\;value\;in\;the\;AR\;group.$$

$$T_{c}:\;the\;value\;in\;the\;sham\;group.$$

**Figure 7:**
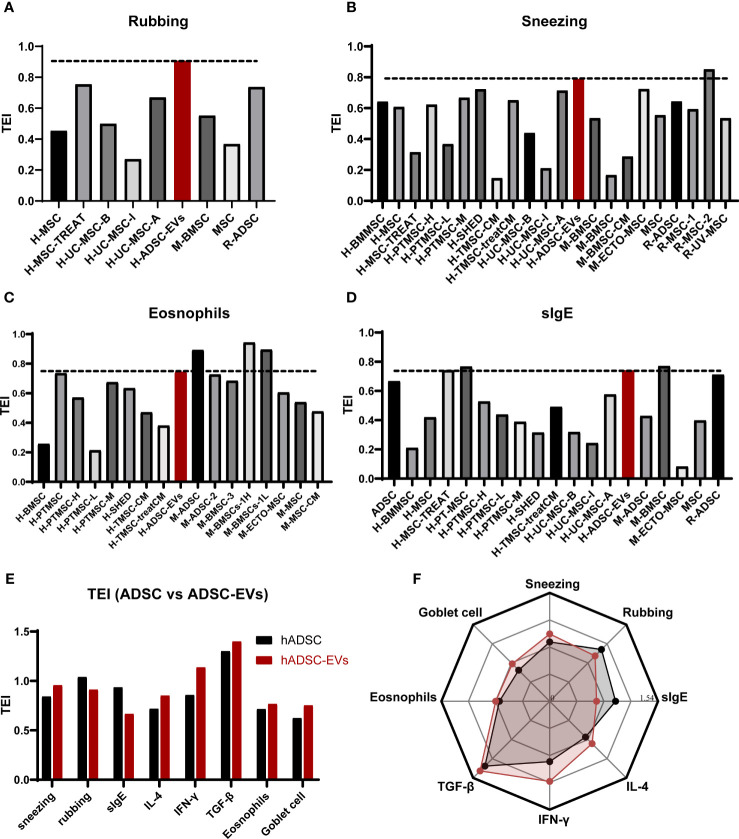
Comparison of therapeutic effect. **(A)** The TEI of rubbing. **(B)** The TEI of sneezing. **(C)** The TEI of eosinophil counts. **(D)** The TEI of serum sIgE levels. **(E)** The TEI of ADSC and ADSC-EVs. **(F)** the radar chart of TEI comparison between ADSC and ADSC-EVs.

To assess the therapeutic effect on sneezing, rubbing, eosinophil levels, and sIgE concentration (**Figures 7A–D**), we quantified the TEI associated with the administration of hADSC-EV and other treatments. The TEI of rubbing in animals treated with hADSC-EV was higher than any other treatment, regardless of the use of homologous or heterologous transplantation. Interestingly, the levels of sneezing and eosinophils indicated by TEI were higher in animals treated with hADSC-EV compared to those treated with heterologous MSC (human-derived) (**Figure 7B** - before the red bar) but lower than those treated with some homologous MSC (mouse-derived) (**Figure 7B** - after the red bar). The concentration of sIgE TEI in animals treated with hADSC-EV was higher than that in animals treated with most heterologous MSC (human-derived) treatments, but lower than that in animals partially treated with homologous MSC (murine-derived) treatments. These results suggest that homologous transplantation may be more effective in treating AR in mice, and homologous EVs may be even more effective.

To further verify that the therapeutic effect of ADSC-EVS is superior to that of ADSC, we performed TEI analysis on the previous data on ADSC treatment of allergic rhinitis in mice and conducted a multidimensional comparison with the data in this study (62). We found that although the TEI of rubbing and serum sIgE in the hADSC-EVs group was lower than that in the ADSC group, the TEI of other indicators in the ADSC-EVs group was higher than that in the ADSC group (**Figures 7E, F**). These results suggest that ADSC-EVS has more effective than ADSC in the treatment of AR mice.

Meanwhile, after comparing the frequency of administration, our study performed only 2 administrations of EVs, which is notably lower than the 3 or 5 administrations reported in the 17 references above. However, our study demonstrated comparable or even superior therapeutic effectiveness in treating AR.

## 4 Discussion

In our research, the findings collectively demonstrate that hADSC-EVs effectively relieved nasal symptoms, including sneezing, nasal rubbing, eosinophil infiltration, and goblet cell hyperplasia. hADSC-EVs significantly reduced serum sIgE production and minimized IL-4 and IFN-γ levels in spleen lymphocytes. Based on the decreased IL-4 and IFN-γ mRNA expression, an immunosuppressive effect at the genetic level was evident. ADSC-EVs promote the secretion of TGF-β1, TGF-β can induce naive CD4+ T cells to differentiate into pTreg cells when stimulated by antigen (19). These data suggested that ADSC-EVs could promote the increase of Treg differentiation. ADSC-EVs decreased the expression of IL-17 mRNA and the proportion of Th17 cells in the spleen, suggesting that ADSC-EVs could inhibit the differentiation and proliferation of Th17 cells (**Figures S3**, **4A–D**). Combined with the upregulation of TGF-β1 secretion, ADSC-EVs can alleviate the Th17/Treg imbalance to some extent (63, 64). Furthermore, hADSC-EVs improved the Th1/Th2 ratio, suggesting the potential for resolving Th1/Th2 imbalances. Our findings indicate that hADSC-EVs mitigate AR symptoms in mice by regulating inflammation through a specific mechanism. These findings agree with prior research on EVs from MSCs *in vitro* (16, 26, 29, 65–69). Additionally, our results indicate no significant difference in the duration of treatment with ADSCs-EVs and the relief of AR symptoms. Although this discovery may be constrained by the experimental design, it shows that the healing effectiveness of ADSC-EVs is relatively sustained and doesn’t necessitate multiple doses, which may be beneficial in preventing any adverse effects caused by multiple dosing.

Interestingly, it was observed that the hADSC-EV groups had significantly lower secretion and mRNA expression of IFN-γ than the AR group. Previous studies on the treatment of a murine rhinitis model have suggested that IFN-γ secretion could increase further after effective treatment, although IFN-γ secretion is significantly higher in AR model mice than in normal mice (45, 47, 55, 70). These studies suggested that the increase in IFN-γ concentration provides evidence of a shift in the Th2 to Th1 immune response to allergens in AR therapy. However, in fact IFN-γ has long been thought to have a pleiotropic effect in autoimmunity (71). IFN-γ production is regulated by natural killer cells (NK), natural killer T cells (NKT), CD8^+^ and CD4^+^ T cells, so IFN-γ is not a definitive marker of TH1differentiation (72, 73).Studies have found that compared to healthy individuals, patients with AR had a higher number of NK cells that secreted type 2 cytokines in their peripheral blood (74). Meanwhile, Treg cells limit IFN-γ secretion to control macrophage accrual and phenotype (75, 76). These results suggest that the secretion of IFN-γ is more beneficial for the treatment and recovery of AR mice. Some studies have found that IFN-γ secretion may not change significantly or may even decrease significantly after effective AR therapy *in vivo* and *in vitro *(26, 29, 67, 77–83). The level of IFN-γ was significantly lower in the conditioned media from the T-MSCs group than in the AR group (58). Based on the above, we suggest that the decrease in IFN-γ levels serves as a marker for successful treatment of AR.

Studies have shown that EVs were also found to inhibit the production of the Th1 inflammatory factors, such as IFN-γ (77). MSC-EVs have been shown to ameliorate inflammatory responses in a variety of diseases by modulating the phenotype of tissue-resident macrophages towards the M2 type. MSC-EVs can inhibit the activation of pro-inflammatory M1 macrophages in favour of pro-resolving M2 macrophages, in parallel with VEGF-A, IFN-γ, IL-12, and TNF-α reduction as well as the upregulation of IL-10 (84, 85). MSC-EVs partially activated the AKT1/AKT2 pathway by attenuating the post-infarction inflammation and cardiomyocyte apoptosis (86). MSC-EVs could also inhibit DC cell maturation and activation (85). MSC-EVs could play a protective and anti-inflammatory role by reducing NK cell recruitment (87, 88). In addition to anti-inflammatory proteins, there are many miRNAs with anti-inflammatory functions in the contents of MSC-EVs (89).

In our study, we demonstrated that hADSC-EVs reduced the levels of IFN-γ and IL-4 in peripheral blood. Compared to the AR group, there was an upregulation of TGF-β1 secretion, a decrease in IL-17 mRNA expression, and a significant reduction in the proportion of Th17 cells. Despite the absence of Treg cell research, our findings indicated that the immunomodulatory mechanism of ADSC-EVs not only rectifies imbalances in Th1/Th2 and Treg/Th17, but also mitigates inflammatory response within the body. The inflammatory regulatory role of ADSC-EVs may be more complex. It may involve inhibition of T cell differentiation, intrinsic lymphocyte regulation, macrophage lymphocyte regulation and even macrophage polarization in inflammatory environments.

Due to the lack of more cytokine index detection and mechanism exploration, we believed that ADSC-EVs have the function of alleviating allergic rhinitis in mice, but the mechanism involved still needs to be further explored. Besides, the study has some limitations that should be optimized. in the absence of original reference data, our results analysis included only TEI results for the mean of the indicators. These data were only used to assess trends in treatment effects and could not be compared using statistical analysis.

## 5 Conclusion

hADSC-EVs had beneficial effects in the ovalbumin-induced AR mice, acting on the inflammatory process (reducing eosinophil percentage and improving goblet cell enlargement in the nasal mucosa; reducing serum levels of sIgE, IL-4, IFN-γ; and improving the immune imbalance of Th1/Th2 cells). The findings imply that hADSC-EVs could be a promising cell-free therapy for allergic diseases, including AR.

## Data availability statement

The original contributions presented in the study are included in the article/**Supplementary Material**. Further inquiries can be directed to the corresponding authors.

## Ethics statement

Ethical approval was not required for the studies on humans in accordance with the local legislation and institutional requirements because only commercially available established cell lines were used. The animal study was approved by Ethics Committee of Experimental Animal Center, Tongji University. The study was conducted in accordance with the local legislation and institutional requirements.

## Author contributions

WY: Data curation, Formal Analysis, Investigation, Methodology, Resources, Software, Validation, Visualization, Writing – original draft, Writing – review & editing. ZP: Data curation, Formal Analysis, Investigation, Methodology, Software, Validation, Visualization, Writing – original draft. JZ: Data curation, Formal Analysis, Investigation, Methodology, Writing – review & editing. LW: Conceptualization, Data curation, Investigation, Methodology, Writing – review & editing. JL: Data curation, Investigation, Methodology, Writing – review & editing. SZ: Conceptualization, Data curation, Formal Analysis, Methodology, Writing – review & editing. ZZ: Conceptualization, Data curation, Investigation, Writing – review & editing. KF: Conceptualization, Data curation, Investigation, Writing – review & editing. DD: Conceptualization, Funding acquisition, Project administration, Resources, Supervision, Writing – review & editing. ZG: Conceptualization, Funding acquisition, Project administration, Resources, Supervision, Validation, Writing – review & editing. SY: Conceptualization, Funding acquisition, Resources, Supervision, Validation, Visualization, Writing – review & editing.
