# Supplementary material for: Extracellular vesicles from adipose stem cells ameliorate allergic rhinitis in mice by immunomodulatory
Source: Front Immunol. 2023 Dec 8;14:1302336. doi: 10.3389/fimmu.2023.1302336 (PMC10739383; doi:10.3389/fimmu.2023.1302336)
Supplement: Supplementary file 1 [file DataSheet_1.docx]

Extracellular vesicles from adipose stem cells ameliorate allergic rhinitis in mice by immunomodulatory

Wenhan Yang ^1,2, #^, Zhiyu Pan ^1, #^, Jiacheng Zhang ^1^, Lian Wang ^2^, Ju lai ^1,2^, Shican Zhou ^1,2^, Zhili Zhang ^1^, Kai Fan ^1^, Dan Deng ^3,4, *^, Zhengliang Gao ^5,6,7, *^, Shaoqing Yu ^1,8, *^

^1^ Department of Otorhinolaryngology Head and Neck Surgery, Tongji Hospital, School of Medicine, Tongji University, 389 Xincun Road, Putuo District, Shanghai, China.

^2^School of Medicine, Tongji University, 500 Zhennan Road, Putuo District, Shanghai, China.

^3^Department of Dermatology, Shanghai Children’s Medical Center, School of Medicine, Shanghai Jiao Tong University, Shanghai, China.

^4^Xinhua Hospital, Shanghai Jiaotong University School of Medicine, Department of Dermatology, Shanghai, China.

^5^Fundamental Research Center, Shanghai YangZhi Rehabilitation Hospital (Shanghai Sunshine Rehabilitation Center), School of Medicine, Tongji University, Shanghai, China.

^6^Shanghai Engineering Research Center of Organ Repair, School of Medicine, Shanghai University, 99 Shangda Road, Shanghai, China.

^7^Institute of Geriatrics (Shanghai University), Affiliated Nantong Hospital of Shanghai University (The Sixth People's Hospital of Nantong), School of Medicine, Shanghai University, Nantong, China.

^8^Department of allergy, Tongji Hospital, School of Medicine, Tongji University, Shanghai, China
^#^ These authors contributed equally to this work and share first authorship.

**Supporting information**

**Supple Table 1.** MSCs in experimental models of allergic rhinitis.

| Animals | Source of MSCs | AR model protocol | Administration and dosage | Related indicators | Ref. |
| --- | --- | --- | --- | --- | --- |
| BALB/c mice | mouse BMSCs | sensitization:  0, 7day  challenge:  14-21days | Administration: 22day  Dosage: 1*10^6^; 2*10^6^ | Number of eosinophil/ mm basal lamina | (1) |
| Mice | ECTO-MSCs derived from mouse nasal mucosa | sensitization:  1, 14day challenge:  21-23days | Administration:  18-20days  Dosage:2*10^6^ | sneeze numbers per hour | (2) |
| BALB/c mice | mice ADSC | sensitization:  0, 14day challenge:  21-23days | Administration:  18-20days  Dosage: 2*10^6^ | Number of eosinophils in the nasal mucosa | (3) |
| Adult male albino rats | ADSC from abdominal wall of rats | sensitization:  1, 5, 10day  challenge:  15-21days | Administration:  15,21,28days  Dosage: 1*10^6^ | sneezing and nose scratching per 10 min | (4) |
| BALB/c mice | ADSC; human palatine tonsil MSC | sensitization:  1, 5, 10day challenge:  15-21days | Administration:  18-23days  Dosage:0.5*10^6^ | Eosinophil infiltration in the nasal mucosa | (5) |
| Sprague‐Dawley rats | humans UC-MSC | sensitization:  1, 5, 10day challenge:  15-21days | Administration:  22day;  22, 29,36,43days  Dosage:0.5*10^6^ | sneeze numbers per 10 minutes | (6) |
| BALB/c mice | mice BMSC | sensitization:  1, 7, 14, 21day challenge:  22-35days | Administration:  22-35days  Dosage: 0.5*10^6^ | sneezing and nasal rubbing per15 min; Eosinophil infiltration in the nasal mucosa | (7) |
| Wistar rats | rats BMMC-conditioned media | sensitization:  1, 8day  challenge:  14-32days | Administration:  33-35days  Dosage:  BSA 500μg/ ml |  | (8) |
| Sprague-Dawley rats | MSC | sensitization:  1, 5, 10day challenge:  15-35days | Administration:  15,21,27,33days  Dosage: 5*10^6^ | sneezing and nose scratching per 10 min | (9) |
| Wistar rats | Rat MSCs | sensitization:  1, 5, 10day challenge:  15-21days | Administration:  22-28days  Dosage: 1*10^6^ | sneezing and nose scratching per 10 min | (10) |
| BALB/c mice | human deciduous teeth | sensitization:  0, 7, 14day challenge:  21-27days | Administration:  18-20days  Dosage: 5*10^5^ | sneezing and nose scratching per 10 min | (11) |
| BALB/c mice | human palatine tonsil | sensitization: 0, 7, 14day challenge:  21-27days | Administration:  14-25days  Dosage: 0.1,10,25mg | sneezing and nose scratching per 15 min | (12) |
| BALB/c mice | human umbilical cord MSC |  | Administration:  22, 25, 28, 30, 33, 37days Dosage: 0.4* 10^5^ | sneezing and nose scratching per 15 min | (13) |
| BALB/c mice | miR-138-5p inhibition modified hMSC | sensitization:  0, 7, 14day challenge:  21-35days | Administration:  21-35days  Dosage: 1*106 | sneezing and nose scratching per 10 min | (14) |
| BALB/c mice | human tonsil tissues MSC | sensitization:  0, 7, 14day  challenge:  23-28days | Administration:  18-23days  Dosage: 20 μL | sneezing and nose scratching per 20 min | (15) |
| BALB/c mice | male BALB/c mice msc | sensitization:  1, 3, 5, 7, 9, 11, 13day challenge:  15-19days | Administration:  20-24days  Dosage: 1*106 | sneezing and nose scratching per 15 min | (16) |
| male Wistar rats | Rats' umbilical-cord MSC | sensitization:  0, 1, 10day challenge:  15-21days | Administration: 22-28days  Dosage: 1*106 | sneezing and nose scratching per 10min | (17) |

**REFERENCES**

1. Işık S, Karaman M, Adan A, Kıray M, Bağrıyanık HA, Sözmen Ş, et al. Intraperitoneal mesenchymal stem cell administration ameliorates allergic rhinitis in the murine model. European archives of oto-rhino-laryngology : official journal of the European Federation of Oto-Rhino-Laryngological Societies (EUFOS) : affiliated with the German Society for Oto-Rhino-Laryngology - Head and Neck Surgery. 2017;274(1):197-207.

2. Yang C, Li J, Lin H, Zhao K, Zheng C. Nasal mucosa derived-mesenchymal stem cells from mice reduce inflammation via modulating immune responses. PloS one. 2015;10(3):e0118849.

3. Cho KS, Park HK, Park HY, Jung JS, Jeon SG, Kim YK, et al. IFATS collection: Immunomodulatory effects of adipose tissue-derived stem cells in an allergic rhinitis mouse model. Stem cells (Dayton, Ohio). 2009;27(1):259-65.

4. Ebrahim N, Mandour YMH, Farid AS, Nafie E, Mohamed AZ, Safwat M, et al. Adipose Tissue-Derived Mesenchymal Stem Cell Modulates the Immune Response of Allergic Rhinitis in a Rat Model. Int J Mol Sci. 2019;20(4).

5. Samivel R, Kim EH, Chung YJ, Mo JH. Immunomodulatory effect of tonsil-derived mesenchymal stem cells in a mouse model of allergic rhinitis. American journal of rhinology & allergy. 2015;29(4):262-7.

6. Li C, Fu Y, Wang Y, Kong Y, Li M, Ma D, et al. Mesenchymal stromal cells ameliorate acute allergic rhinitis in rats. Cell biochemistry and function. 2017;35(7):420-5.

7. Zhao N, Liu Y, Liang H, Jiang X. Bone marrow-derived mesenchymal stem cells reduce immune reaction in a mouse model of allergic rhinitis. American journal of translational research. 2016;8(12):5628-36.

8. Keyhanmanesh R, Rahbarghazi R, Aslani MR, Hassanpour M, Ahmadi M. Systemic delivery of mesenchymal stem cells condition media in repeated doses acts as magic bullets in restoring IFN-γ/IL-4 balance in asthmatic rats. Life Sci. 2018;212:30-6.

9. Fu Y, Kong Y, Li J, Wang Y, Li M, Wang Y, et al. Mesenchymal stem cells combined with traditional Chinese medicine (qi-fang-bi-min-tang) alleviates rodent allergic rhinitis. J Cell Biochem. 2020;121(2):1541-51.

10. Restimulia L, Ilyas S, Munir D, Putra A, Madiadipoera T, Farhat F, et al. The CD4+CD25+FoxP3+ Regulatory T Cells Regulated by MSCs Suppress Plasma Cells in a Mouse Model of Allergic Rhinitis. Medical archives (Sarajevo, Bosnia and Herzegovina). 2021;75(4):256-61.

11. Dai YY, Ni SY, Ma K, Ma YS, Wang ZS, Zhao XL. Stem cells from human exfoliated deciduous teeth correct the immune imbalance of allergic rhinitis via Treg cells in vivo and in vitro. Stem cell research & therapy. 2019;10(1):39.

12. Park IS, Kim JH, Bae JS, Kim DK, Mo JH. The Supernatant of Tonsil-Derived Mesenchymal Stem Cell Has Antiallergic Effects in Allergic Rhinitis Mouse Model. Mediators of inflammation. 2020;2020:6982438.

13. Kan XL, Pan XH, Zhao J, He J, Cai XM, Pang RQ, et al. Effect and mechanism of human umbilical cord mesenchymal stem cells in treating allergic rhinitis in mice. Sci Rep. 2020;10(1):19295.

14. Tang H, Han X, Li T, Feng Y, Sun J. Protective effect of miR-138-5p inhibition modified human mesenchymal stem cell on ovalbumin-induced allergic rhinitis and asthma syndrome. J Cell Mol Med. 2021;25(11):5038-49.

15. Park IS, Kim DK, Kim JH, Bae JS, Kim EH, Yoo SH, et al. Increased Anti-Allergic Effects of Secretome of Low-Level Light Treated Tonsil-Derived Mesenchymal Stem Cells in Allergic Rhinitis Mouse Model. American journal of rhinology & allergy. 2022;36(2):261-8.

16. Zou W, Zou P, Zhang J, Cai X, Mao X, Liu G. Conditioned medium from the bone marrow mesenchymal stem cells modulates immune response via signal transduction and activator of transcription 6 signaling pathway in an allergic rhinitis mouse model. Allergologia et immunopathologia. 2022;50(4):105-14.

17. Restimulia L, Ilyas S, Munir D, Putra A, Madiadipoera T, Farhat F, et al. Rats' umbilical-cord mesenchymal stem cells ameliorate mast cells and Hsp70 on ovalbumin-induced allergic rhinitis rats. Medicinski glasnik : official publication of the Medical Association of Zenica-Doboj Canton, Bosnia and Herzegovina. 2022;19(1).

**Supplementary figure**

**
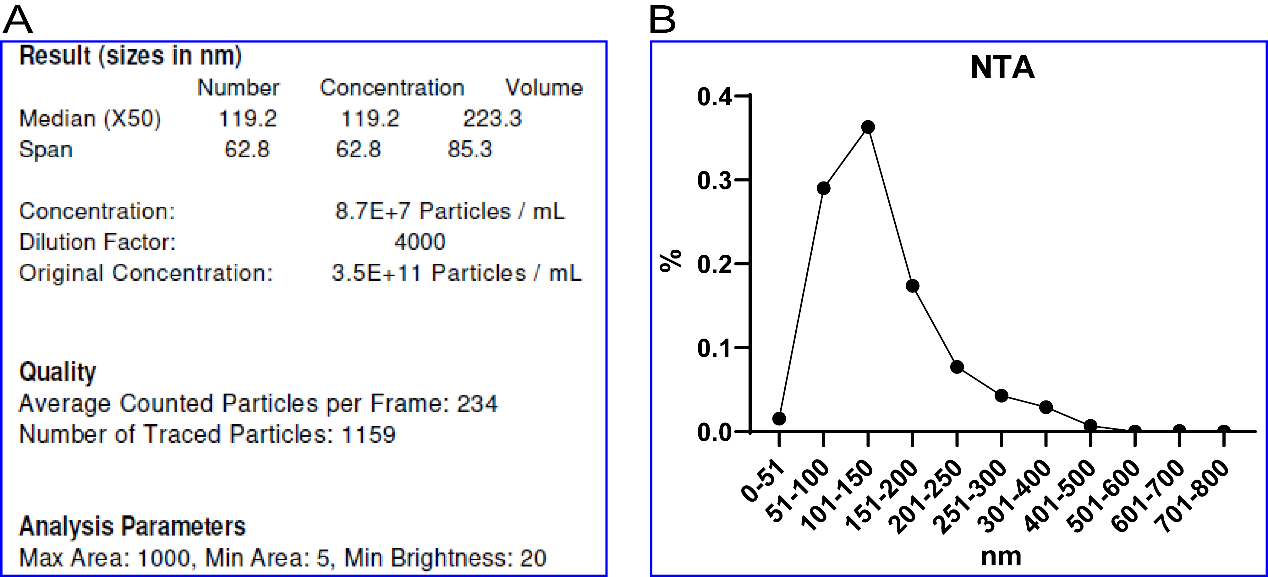
**

**Supple Figure 1.** Particle size distribution of EVs measured by nanoparticle tracking analysis (NTA) **A** particle concentration. **B** Percentage of particles within the size range.


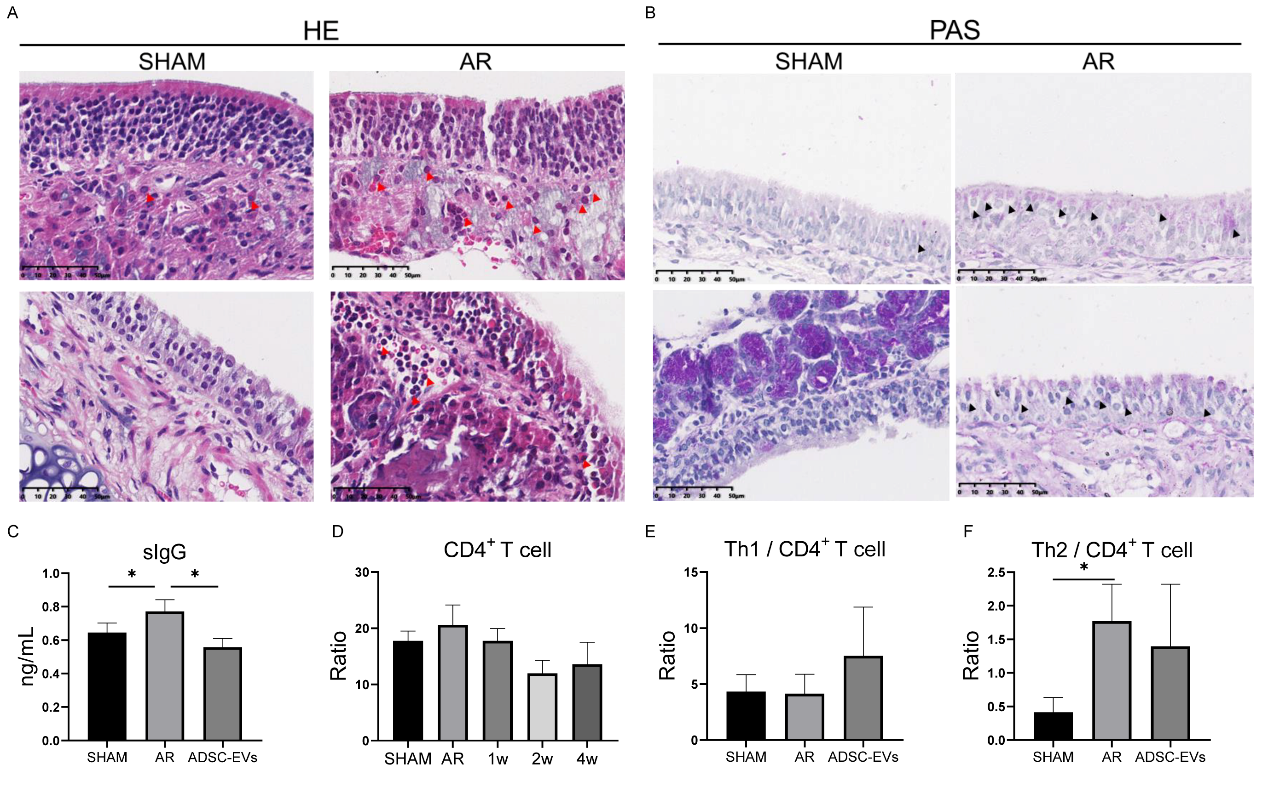


**Supple Figure 2.** Nasal mucosa section, the secretion of antigen-specific antibodies and cytokine and flow cytometry analysis. **A** H&E staining of the nasal mucosa. **B** PAS staining of the nasal mucosa. **C** Concentration of sIgG measured. **D-F** the count of CD4+T cell, Th1 cell, and Th2 cell. Data are expressed as mean ± SD, n=8 or 24, *P < 0.05, **P < 0.01, ***P < 0.001.

**
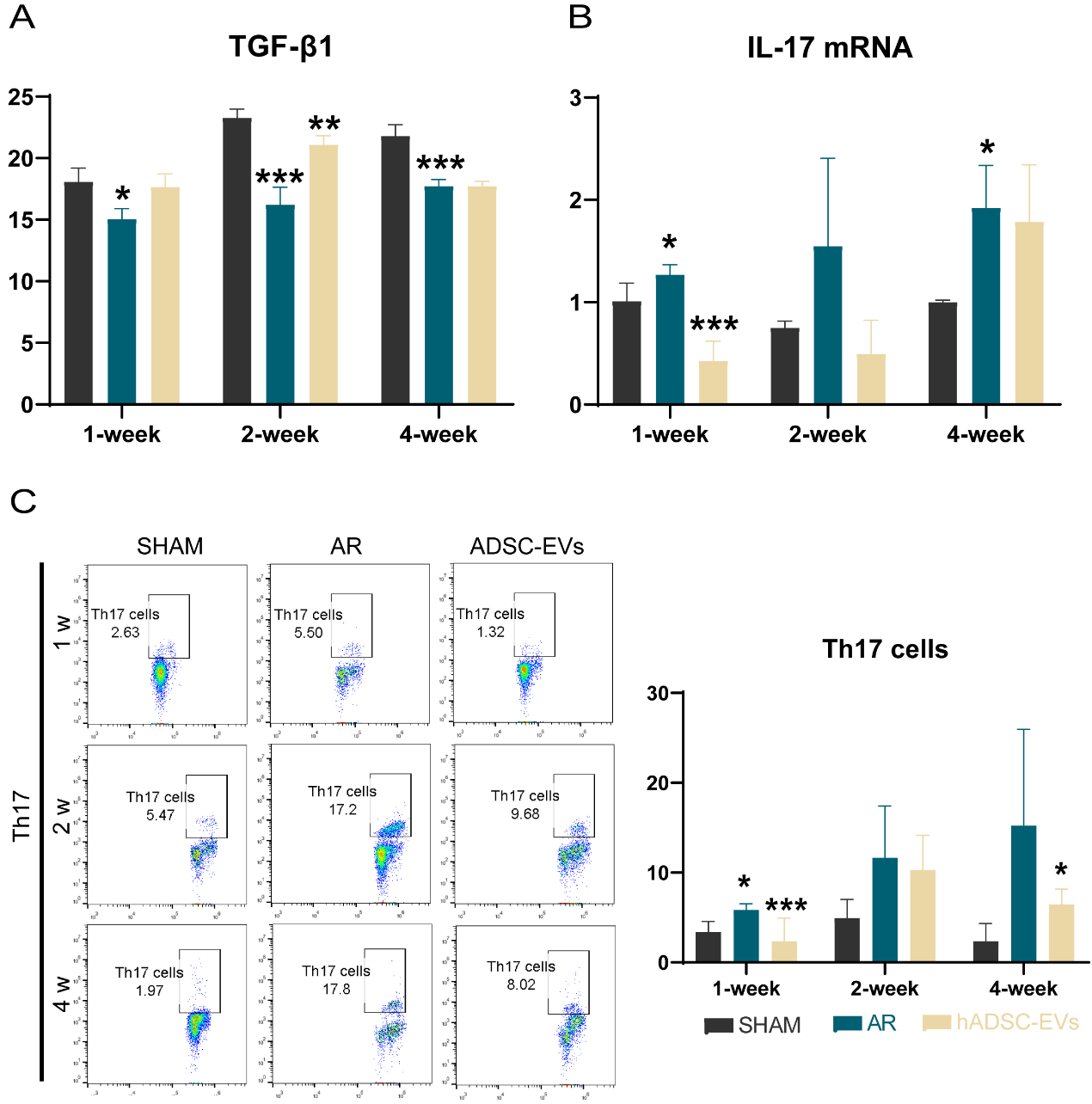
Supple Figure 3.** hADSC-EVs alleviated the Th17 imbalance **A** Concentration of TGF-β in the serum measured by ELISA. **B** Relative expression of IL-17 by qRT‒PCR. **C** The ratio of Th17 cells calculated according to the results from D. **C** Flow cytometry analysis of Th17 cells in splenic lymphocytes. Data are expressed as mean ± SD, n=8, *P < 0.05, **P < 0.01, ***P < 0.001.

**
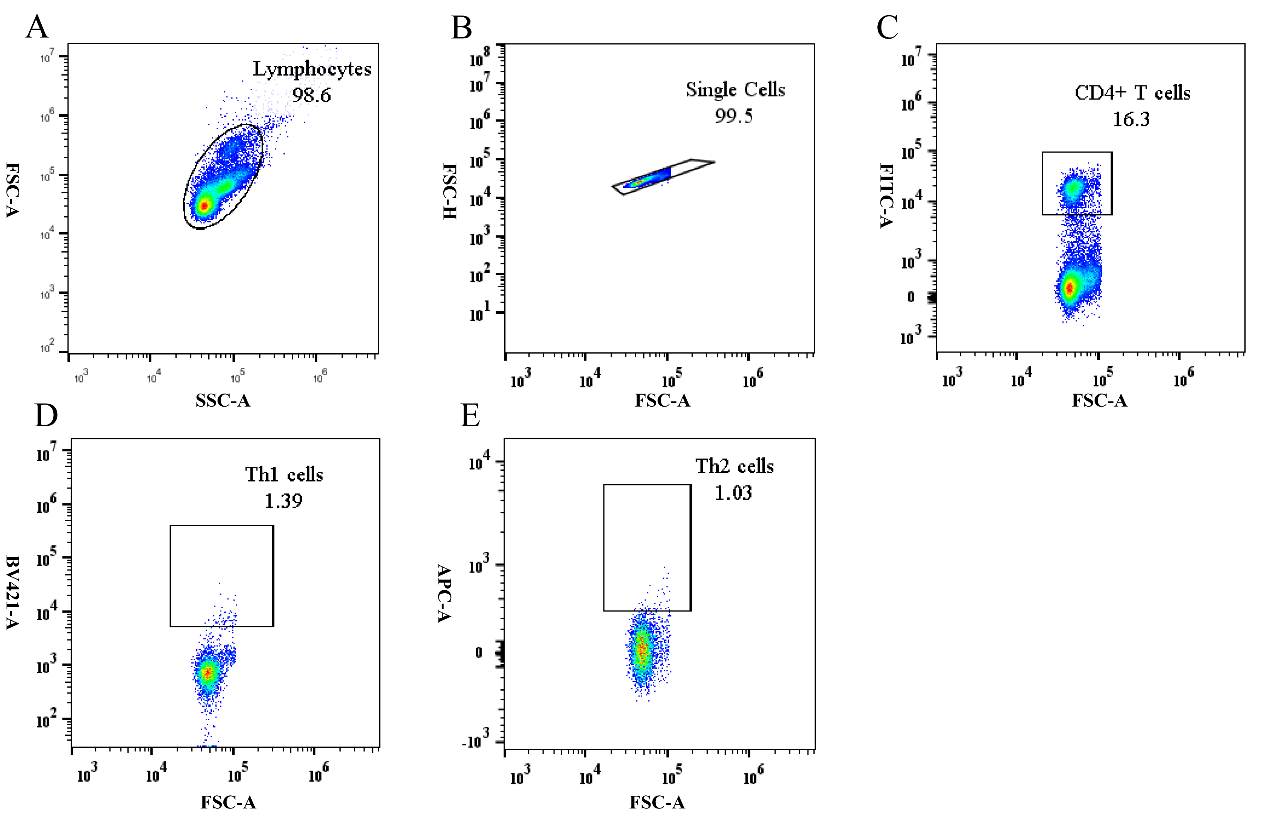
**

**Supple Figure 4.** The gating strategy of the FACS **A** Lymphocytes in total cells. **B** Cell adhesion. **C** CD4^+^ T cells in lymphocytes. **D-E** CD4^+^ Th1 and Th2 cells.


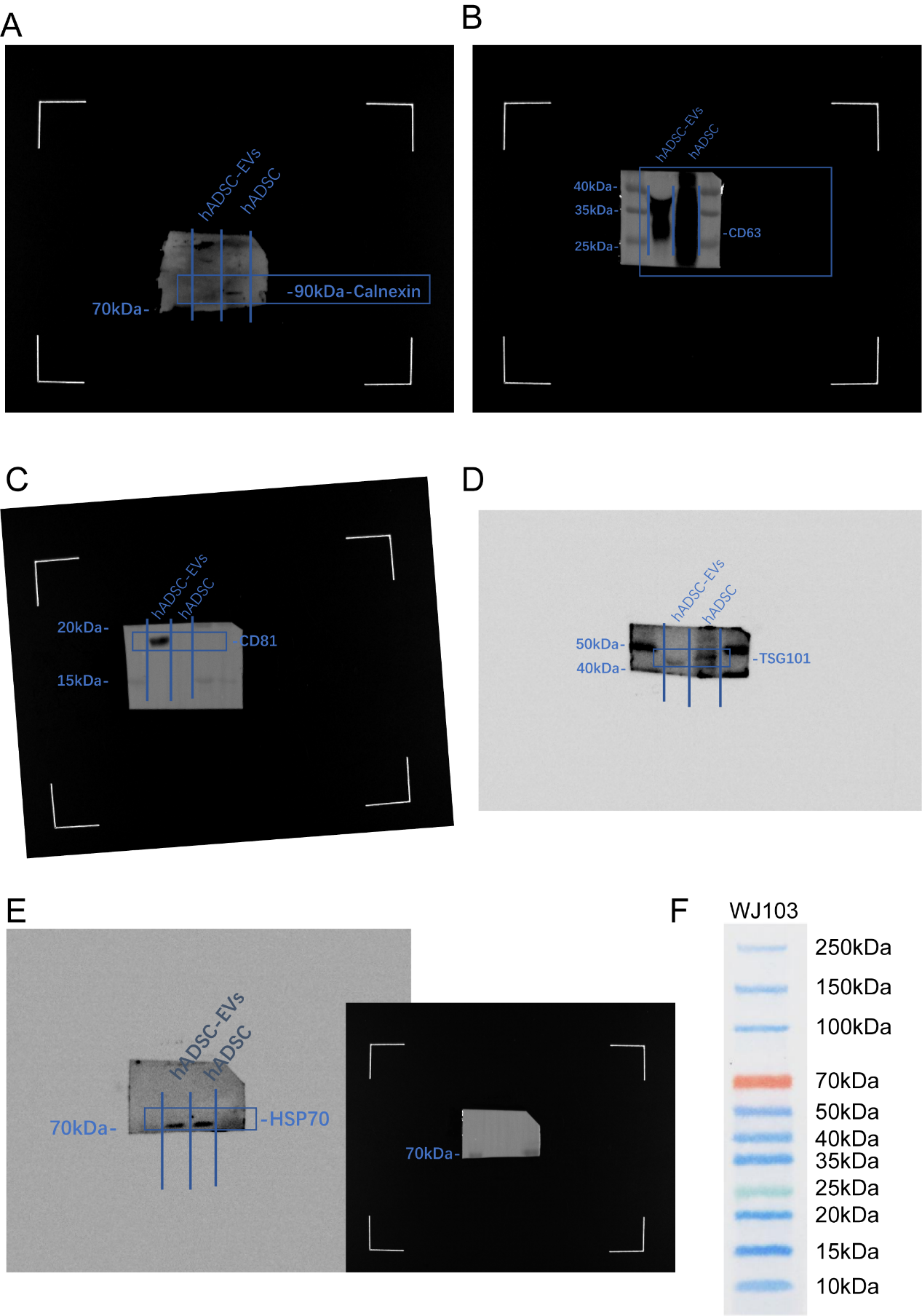


**Supple Figure 5.** The original gel/blot images are displayed below. **A** Calnexin. **B** CD63. **C** CD81. **D** TSG101. **E** HSP70. **F** the marker of western blot.
